# Transcriptomic study reveals changes of lncRNAs in PBMCs from HIV-1 patients before and after ART

**DOI:** 10.1038/s41598-023-49595-z

**Published:** 2023-12-15

**Authors:** Yingying Zhou, Yuqing Huang, Xiaoping Chen, Tielong Chen, Wenjia Hu, Wei Hou, Qi Zhang, Yong Xiong

**Affiliations:** 1 Department of Infectious Diseases, Zhongnan Hospital of Wuhan University, No. 169, Donghu Road, Wuchang District, Wuhan, 430071 Hubei Province China; 2 State Key Laboratory of Virology, Modern Virology Research Center, College of Life Sciences, Wuhan University, Wuhan, 430071 Hubei Province China; 3 State Key Laboratory of Virology/Institute of Medical Virology/Hubei Province Key Laboratory of Allergy and Immunology, School of Basic Medical Sciences, Wuhan University, 185 Donghu Road, Wuhan, 430071 Hubei Province China

**Keywords:** Clinical microbiology, Virus-host interactions, Diseases, HIV infections

## Abstract

Long noncoding RNAs (lncRNAs) play important roles in regulating HIV-1 infection and virus-host interactions. However, it is unclear whether and how ART alters lncRNAs in HIV-infected patients. In the present study, we investigated changes of lncRNAs in PBMCs from HIV-1 patients pre- and post-ART. We identified a total of 974 lncRNAs whose expression was restored to normal levels after ART. Cis-acting analysis showed that six lncRNAs have cis-regulated target genes, among which RP11-290F5.1 and interferon regulatory factor 2 (IRF2) were reported to promote HIV replication. Furthermore, we found that lncRNA CTB-119C2.1, which regulates most mRNAs with differential expression in PBMCs from HIV-1 infected patients after ART, was significantly upregulated by RNA-seq and qRT-PCR assays. KEGG analysis of CTB-119C2.1-associated genes revealed that most of the genes are involved in the p53 signaling pathway and pathways related to cell cycle and DNA replication. Our findings thus reveal the dynamic change of lncRNAs in people living with HIV-1 pre- and post-ART and warrant further investigation of the role of lncRNAs in HIV-1 pathogenesis and treatment.

## Introduction

Human immunodeficiency virus (HIV) is the pathogen of Acquired immunodeficiency syndrome (AIDS). According to the World Health Organization, approximately 37.9 million people around the world were infected with HIV by the end of 2018, and AIDS remains one of the biggest threats to global health.

As a retrovirus, HIV’s life cycle is marked by its single-stranded RNA genome, which replicates through reverse transcription and can integrate into the host genome^1^. Effective replication of HIV requires host cell activation, which leads to increased efficiency in reverse transcription, integration, and viral gene expression, a result of the stimulation of host factors involved in HIV replication^2^. These host factors include cell receptors for viral infection, the proteins responsible for the integration of the viral genome, and the maturation of viral particles. The main treatment for AIDS now focuses on antiretroviral therapy. Antiretroviral therapy (ART) has significantly reduced AIDS-related morbidity and mortality^3^. Although ART has prolonged the survival time of patients, it has not led to a cure for AIDS or a complete elimination of the virus^4^. In recent years, HIV-1 cure has been achieved in several patients by CCR5Δ32/Δ32 allogeneic hematopoietic stem cell transplantation (allo-HSCT)^5–7^. However, this treatment strategy may be difficult to promote because of the high risk of allo-HSCT.

Long noncoding RNA (lncRNA) is a general term for noncoding RNA whose transcriptional length is greater than 200 nucleic acids^8^. They are mainly distributed in the nucleus and cytoplasm, with tissue specificity. These lncRNAs usually do not encode proteins. They participate in a variety of biological processes, such as cell differentiation, canceration, and immune response, and play an important role in the occurrence and prevention of certain diseases^9^. LncRNAs regulate gene expression, chromatin organization, cell transport, RNA decay, and protein translation and affect protein second-generation localization, function, decay, and turnover^10^. Studies have shown that after viral infection, intracellular lncRNAs can regulate viral replication, cellular metabolism, and the immune response^11^. In the study of HIV-1 infection, lncRNAs can control the number of in vivo CD4+ T cells, the apoptosis of infected cells, the life cycle of the virus, and the incubation period of the virus^12^. The development of targeted molecular therapies that target lncRNAs involved in viral replication and pathogenesis may be of great significance to the treatment of AIDS^13,14^. There are still great opportunities and challenges regarding the role of lncRNAs in AIDS progression, which need more comprehensive and in-depth studies.

In the treatment of HIV-1 patients, despite the use of ART, patients continue to experience chronic immune activation and inflammation, which may lead to an increased risk of non-AIDS comorbidities such as metabolic syndrome and cardiovascular disease^15^. Although viral replication is suppressed after ART, the biomarkers of immune activation, inflammation, and clotting disorders do not fully restore to normal, and sustained immune activation is a leading cause of non-HIV complications^16^. At present, there is no effective method of controlling non-HIV complications and simultaneously improving prognosis after antiviral therapy. The accumulating results of lncRNA research in HIV-1 infection suggest a potential role of lncRNAs in clinical HIV-1 infection treatment. In this study, we identified a few lncRNAs involved in HIV-1 replication and pathogenesis. These findings could help to explain the molecular mechanism of the progression of AIDS and provide potential key molecules for targeted molecular therapy in the treatment of HIV-1 infection.

## Results

### Clinical characteristics of the volunteers

In the healthy control group, six healthy volunteers undergoing physical examination in the Physical Examination Center of Zhongnan Hospital were recruited and labeled as H1, H2, H3, H4, H5, and H6. The treatment group consisted of 6 HIV-infected patients who were diagnosed at the AIDS Center of Zhongnan Hospital of Wuhan University and labeled as B1, B2, B3, B4, B5, and B6 before treatment, and as A1, A2, A3, A4, A5, and A6 after treatment. The age, sex, CD4+ and CD8+ T cell counts, plasma viral load, and ART regimen of each participant are shown in Table 1. All 6 HIV-infected subjects' viral loads were below the threshold of detection (< 20 HIV RNA copies/ml) at the timepoint of sampling.

**Table 1:** Subjects' age, sex, CD4+ T lymphocyte count, CD8+ T lymphocyte count, plasma viral load, ART regimen and duration.

Label	Gender	Age	Detection time	CD4+ T lymphocyte (count/µl)	CD8+ T lymphocyte (count/µl)	Plasma viral load (copies/ml)	ART regiment	ART duration (month)
H1	Female	24	2019/12/17					
H2	Female	25	2019/12/17					
H3	Female	25	2019/12/17					
H4	Male	20	2019/12/17					
H5	Female	25	2019/12/17					
H6	Female	24	2019/12/17					
B1	Male	30	2019/4/2	61	699	13,799	TDF + 3TC + EFV	
A1	Male	–	2019/9/29	112	900	< 20		6.0
B2	Male	29	2019/4/17	236	1106	26,853	TDF + 3TC + EFV	
A2	Male		2019/7/15	321	666	< 20		3.0
B3	Male	27	2019/4/19	266	261	96,164	TDF + 3TC + EFV	
A3	Male		2019/10/15	366	550	< 20		6.0
B4	Male	31	2019/4/29	393	504	4704	TDF + 3TC + EFV	
A4	Male		2019/10/28	648	660	< 20		6.0
B5	Male	19	2019/4/29	400	590	5974	TDF + 3TC + EFV	
A5	Male		2019/8/15	642	763	< 20		4.5
B6	Male	48	2019/5/16	283	519	38,386	TDF + 3TC + EFV	
A6	Male		2019/8/14	310	255	< 20		3.0

### Identification of differentially expressed mRNAs and lncRNAs between the ART treatment group and the healthy group

**Figure 1:**
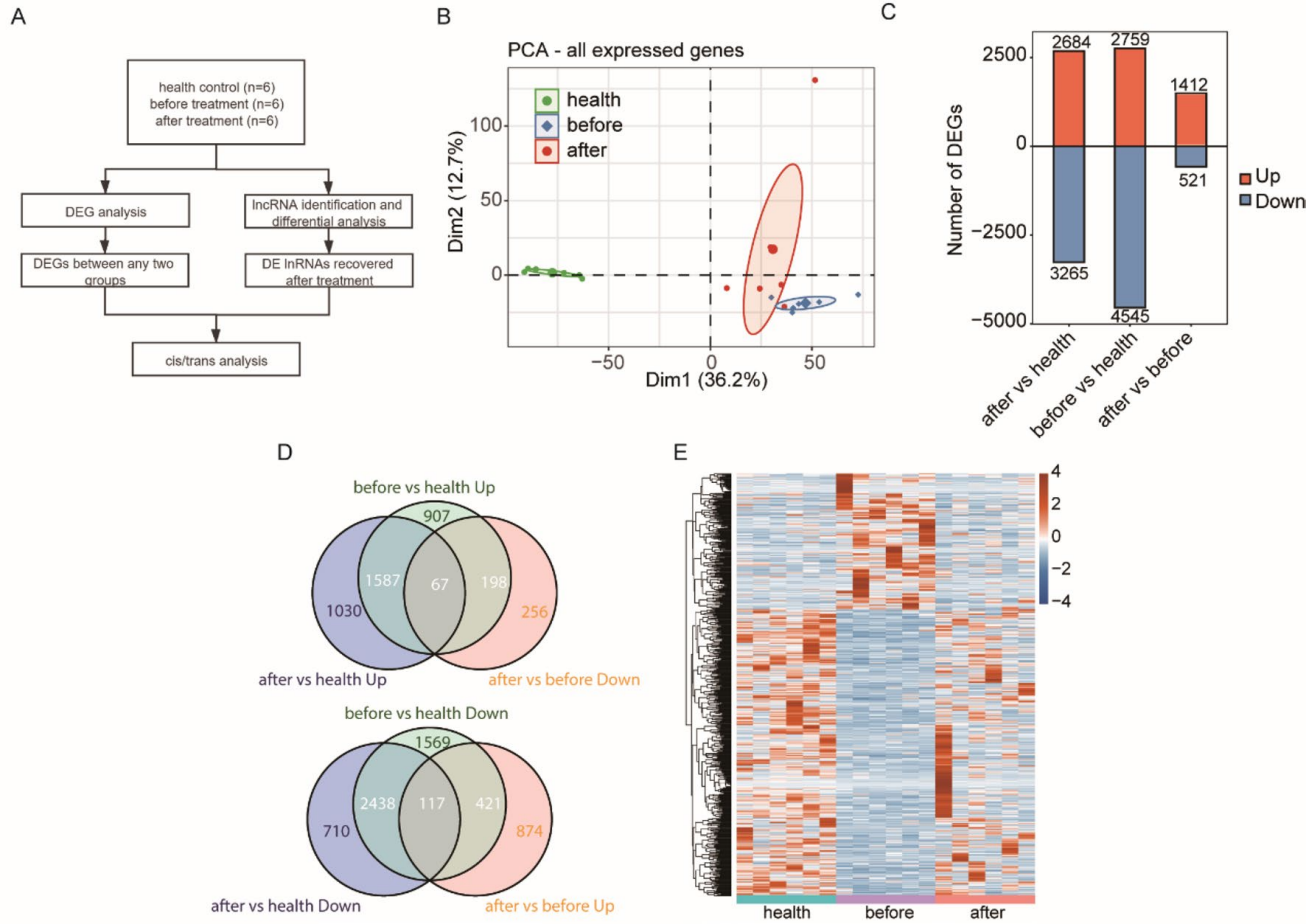
After treatment, the expression levels of many genes showed a recovering tendency to the level of healthy people. (**A**) Illustration of the experimental design and bioinformatics analysis pipeline for this study; (**B**) principal component analysis (PCA) of samples from three groups based on the normalized expression level of all expressed genes. The samples were grouped by disease and treatment state; (**C**) the number of DEGs among different groups. The numbers of upregulated and downregulated DE lncRNAs are shown in a bar plot. (**D**) Venn plot showing differentially expressed genes between treatment samples and healthy controls samples, and between after- and before treatment. Up: upregulated; Down: downregulated; (**E**) Heatmap showing the expression profile of restored genes after treatment, which is 198 genes and 421 genes in (**D**).

To determine how ART changes transcriptomics in PBMCs, we collected PBMCs from 6 HIV-1 infected patients before and after ART and performed RNA-seq. PBMCs from 6 healthy controls were also included as controls. The mRNAs and lncRNAs responding to HIV-1 infection and ART treatment were identified by pairwise comparison among the three groups, and then the functions of the lncRNAs and their regulatory genes were further determined (Fig. 1A). Principal component analysis (PCA) based on all the expressed genes showed high intra-replica reproducibility, and individual samples clustered according to HIV-1 infection and ART (Fig. 1B). Then, we pairwise compared the three groups of samples and obtained genes with significantly upregulated and downregulated expression between groups (Fig. 1C). The results showed that compared to healthy controls, HIV-1 infection significantly up-regulated 2759 genes and down-regulated 4545 genes in PBMCs. ART treatment partially restored gene expression, with 1412 genes showing upregulation and 521 genes showing downregulation. However, there were still2684 upregulated genes and 3265 downregulated genes in PBMCs in ART samples in comparison with healthy controls.

Veen diagram showed that among the 2759 upregulated genes after HIV-1 infection, 198 genes were restored to normal expression, while 67 genes were significantly decreased but still higher than those of healthy controls after ART treatment. Additionally, among the 4545 downregulated genes after HIV-1 infection, 421 genes were restored to normal expression, while 117 genes expressions were upregulated but still lower than those of healthy controls after ART treatment (Fig. 1D). Heatmap analysis further confirmed that those 198 and 421 genes were restored to normal after ART treatment (Fig. 1E).

### ART does not fully restore HIV-1 induced inflammation and post-infection damage in PBMCs

**Figure 2:**
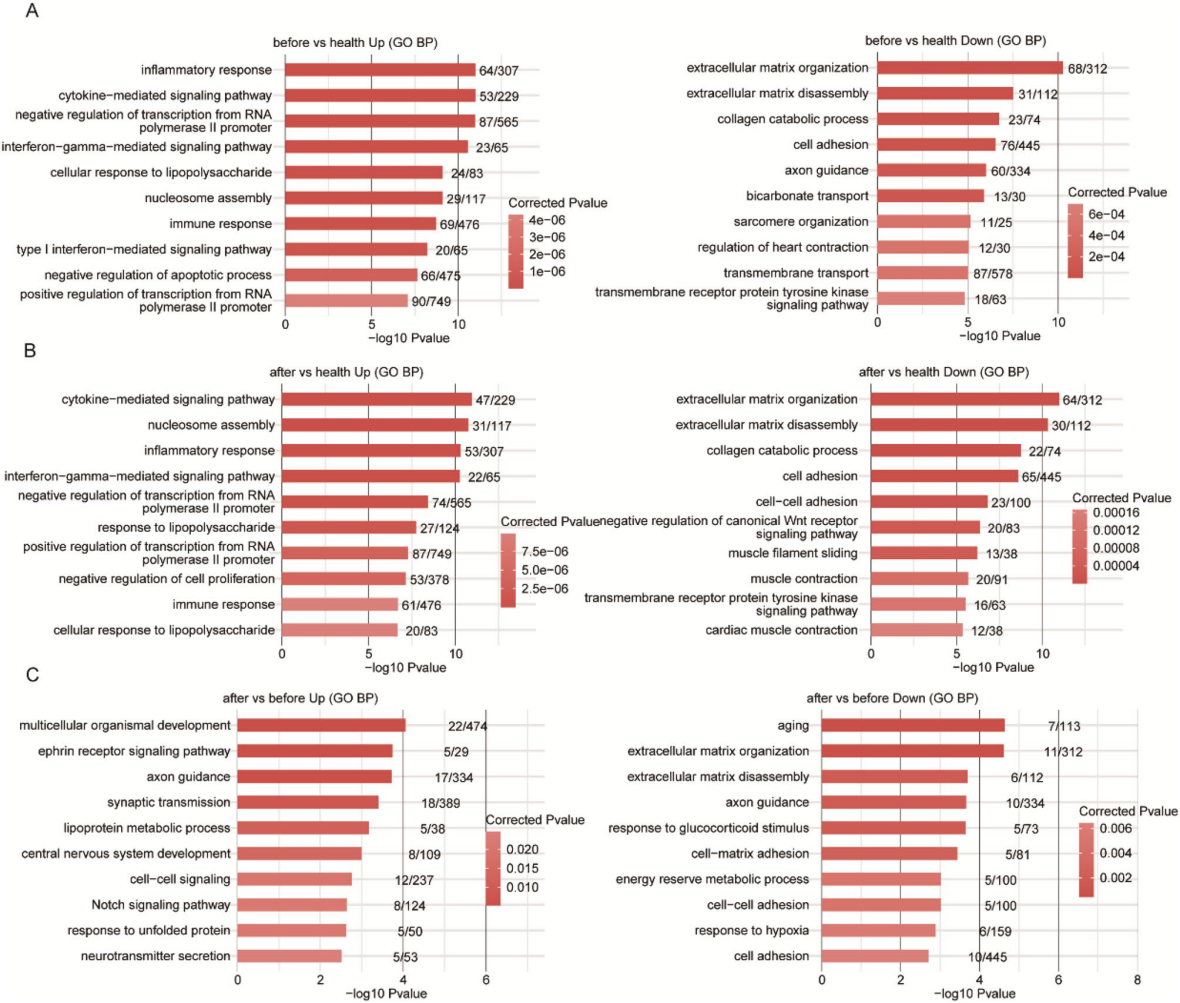
Gene Ontology biological process. (**A**) Top 10 GO biological process terms enriched by differentially expressed genes compared between the before and healthy groups. (**B**) Top 10 GO biological process terms enriched by differentially expressed genes compared between the after and healthy groups. (**C**) Top 10 GO biological process terms enriched by differentially expressed genes compared between the after and before groups.

We next performed gene ontology analysis to determine how HIV-1 infection and ART change the biological process in PBMCs. In HIV patients, most upregulated genes were involved in biological processes related to inflammation/immune response, while most downregulated genes were involved in biological processes related to extracellular matrix organization and cell adhesion **(**Fig. 2A**)**. ART treatment did not fully restore the biological processes involved in inflammatory response extracellular matrix organization in PBMCs since those processes were still upregulated or downregulated compared to PBMCs from healthy controls **(**Fig. 2B,C**)**.

### Features of lncRNAs expression profiling

**Figure 3:**
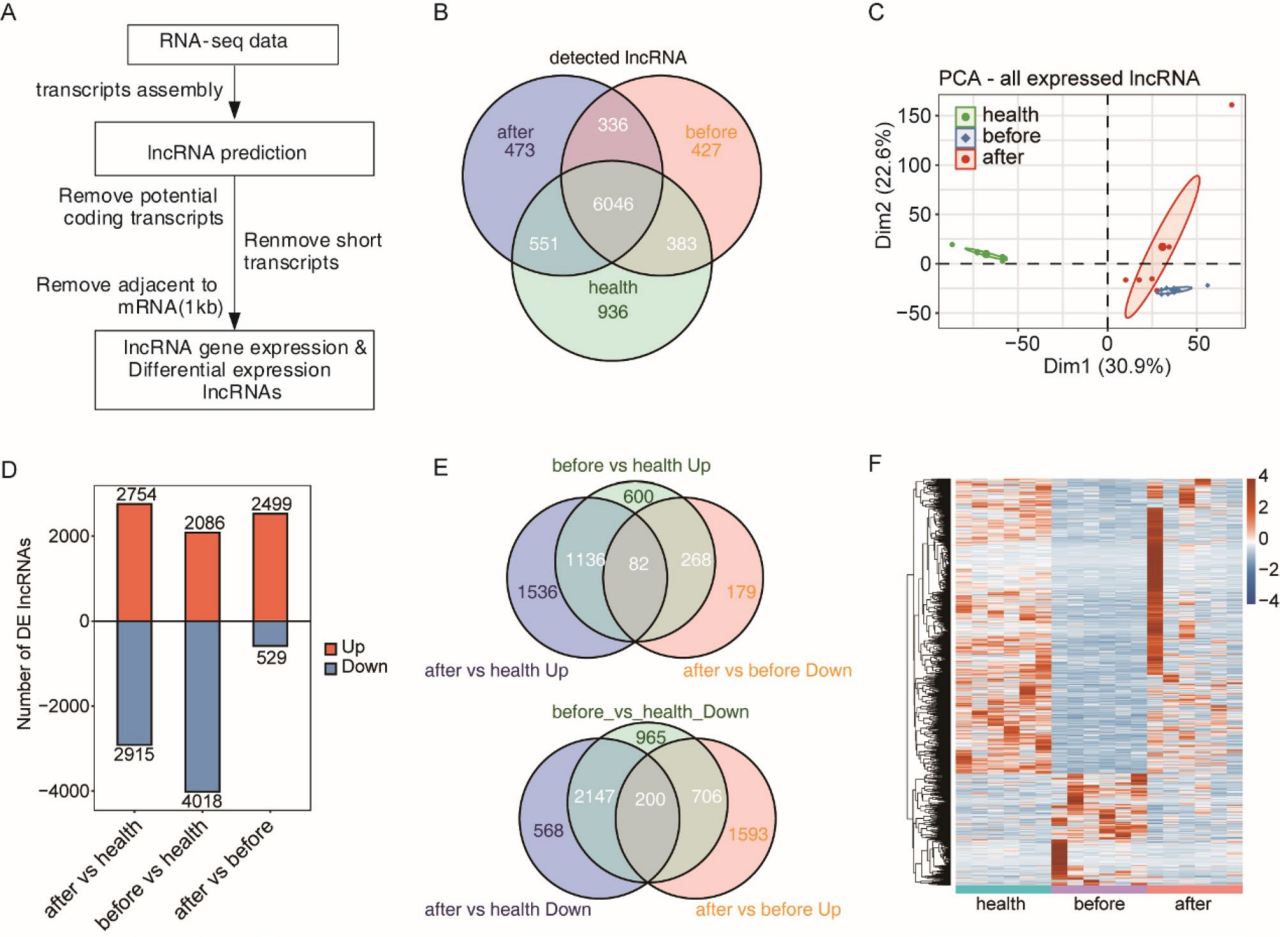
A comprehensive catalog of lncRNAs whose expression levels recovered to no significant difference from those of healthy controls after therapy. (**A**) Illustration of the experimental design and bioinformatics analysis pipeline for the identification of lncRNA genes expressed in samples of three groups. (**B**) Venn diagram of detected lncRNAs in samples of three groups. The lncRNAs that were detected (RPKM ≥ 0.2) in at least two samples of any group were included in this analysis. (**C**) Principal component analysis (PCA) of samples from three groups based on the normalized expression level of all expressed lncRNAs. The samples were grouped by disease and treatment state. (**D**) The number of DE lncRNAs among different groups. The number of upregulated and downregulated DE lncRNAs is shown in a bar plot. (**E**) Venn plot showing differentially expressed lncRNAs between treatment samples and healthy samples and between after- and before treatment. Up: upregulated; Down: downregulated. (**F**) Heatmap showing the expression profile of restored lncRNAs after treatment, which is 268 genes and 706 genes in (**E**).

We further identified and classified lncRNAs (Fig. 3A) in PBMCs from healthy controls and HIV-1 patients before and after ART. A total of 6046 lncRNAs including genome-annotated and predicted lncRNAs, were identified in all three groups of samples (Fig. 3B). Principal component analysis (PCA) of all lncRNA expression showed that the healthy group and the disease group were distinct from each other, indicating that expression of lncRNAs was significantly changed after HIV-1 infection and ART treatment (Fig. 3C). This suggests that lncRNAs may play a significant role in HIV-1 infection. Then, we analyzed the lncRNAs whose expression levels were significantly upregulated or downregulated during HIV-1 infection or treatment (Fig. 3D). Among all identified lncRNAs, 268 abnormally upregulated lncRNAs were restored to normal levels after ART treatment, while 82 abnormally upregulated lncRNAs was decreased but remained higher than those of the healthy group. Correspondingly, 706 abnormally downregulated lncRNAs were restored to normal levels after ART treatment, while 200 abnormally downregulated lncRNAs increased but remained lower than those of the healthy group (Fig. 3E). Finally, we found that a total of 974 lncRNAs showed a tendency to recover to the level of healthy people after treatment (Fig. 3F).

### Exploration of the potential roles of lncRNAs through *trans*- and *cis*-acting analysis

**Figure 4:**
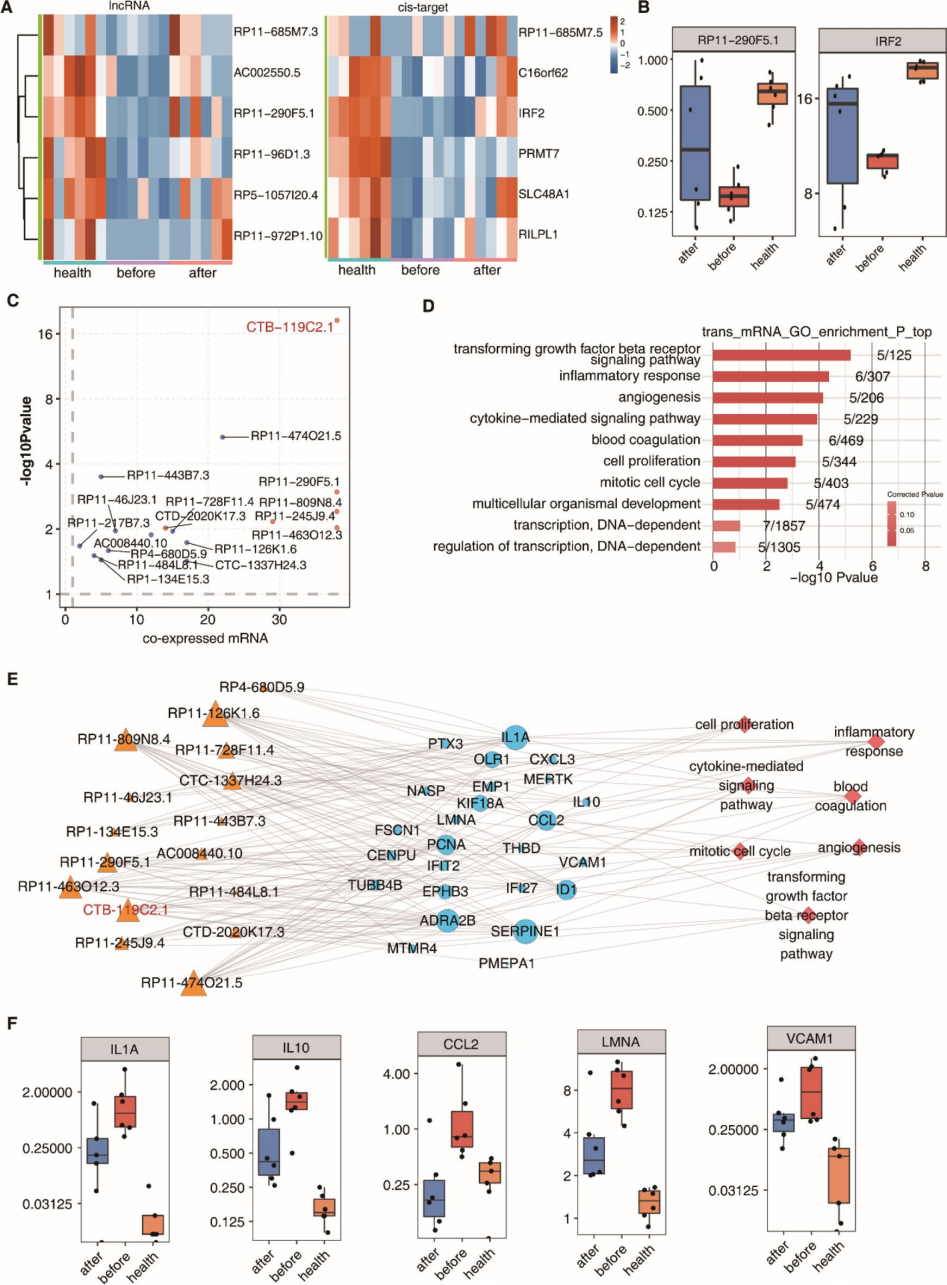
Trans- and cis-acting analysis of lncRNAs whose expression levels recovered to no significant difference with healthy people after therapy. (**A**) Heatmap showing the cis-acting pairs of restored lncRNAs after treatment (left) and target genes (right). (**B**) Boxplots showing the expression status of lncRNA RP11–290F5.1 and its cis-target IRF2 in the healthy, before, and after groups. (**C**) Scatter plot shows DE lncRNAs by after-treatment compared with before-treatment samples and the number of coexpressed DE genes. Red points denote upregulated lncRNAs involved in coexpression pairs, and blue points denote downregulated lncRNAs. Cutoffs of p value < 0.01 and Pearson coefficient ≥ 0.6 were applied to identify the coexpression pairs. Low-abundance genes were filtered out when the expression of more than 20% of genes was less than 0.5. (**D**) GO biological process terms enriched by trans-target genes. (**E**) The coexpression network between DE lncRNAs and DE genes that are involved in the top 7 GO terms shown in (**D**). LncRNAs are on the left, coexpressed genes are in the center, and the gene-enriched GO terms are on the right. (**F**) Boxplots showing the expression status of five trans-target genes from (**E**) in the healthy, before, and after groups.

We analyzed the target genes of 974 lncRNAs, whose expression was restored to that of healthy people after ART treatment. First, we investigated the expression relationship between the restored lncRNAs and their associated genes. According to the cis-regulation analysis, six lncRNAs with homeopathic regulation target genes were identified (Fig. 4A), one of which is lncRNA RP11–290F5.1 (Fig. 4B), whose target gene IRF2 was reported to be related to HIV infection. Then, we analyzed the differential expression of lncRNAs before and after ART treatment by co-expression analysis (Fig. 4C). We identified that lncRNA CTB-119C2.1, with the most obvious expression difference, was the lncRNA with the largest number of associated differentially expressed genes. Based on the results of co-expression, GO enrichment analysis was performed on these genes (Fig. 4D). The top biological processes involved in a series of complications caused by HIV-1 infection are the inflammatory response and cytokine-mediated signaling pathways. The regulatory network among differentially expressed lncRNAs, coexpressed genes, and associated biological processes during HIV-1 infection and treatment was constructed (Fig. 4E). Among the lncRNA-associated genes, expression of IL-1α, IL-10, CCL2, LMNA, and VCAM1, which were reported to be associated with HIV-1 infection, were shown in Fig. 4F.

### Real-time quantitative PCR validation

**Figure 5:**
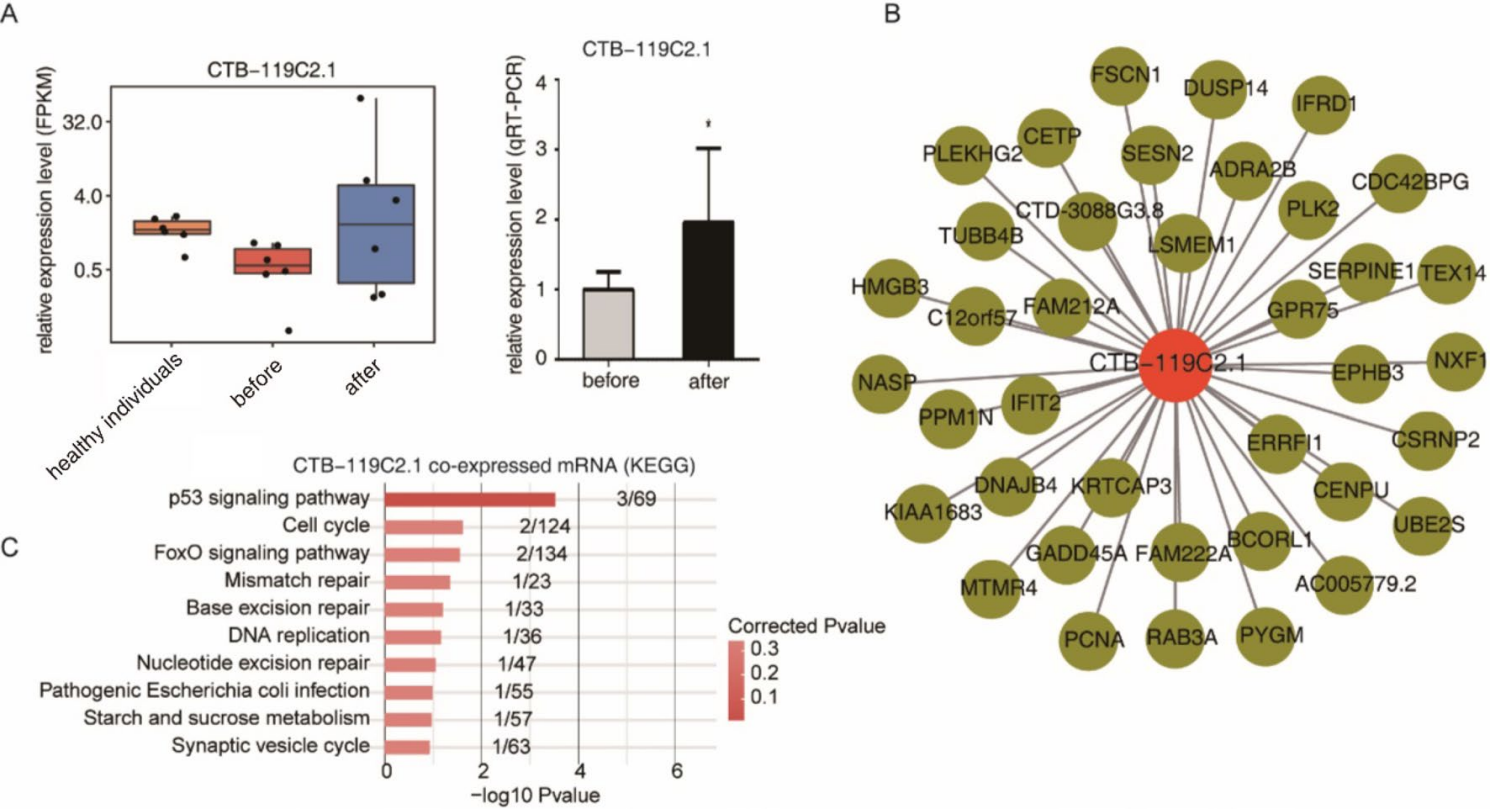
Validation of differential expression of lncRNA CTB-119C2.1 before and after samples and analysis of coexpressed genes. (**A**) Bar plots showing the relative expression level of CTB-119C2.1 using RNA-seq data (left) and qRT–PCR (right) in the before and after groups. (**B**) Coexpressed DE genes of CTB-119C2.1. (**C**) Bar plot showing the top 10 enriched KEGG pathways from coexpressed genes of CTB-119C2.1.

8 HIV-1 infected patients were recruited for the validation study, labeled as b1, b2, b3, b4, b5, b6, b7 and b8 before treatment, and as a1, a2, a3, a4, a5, a6, a7 and a8 after treatment (Table 2). We next validated the lncRNAs mentioned above, which were predicted to play important roles during HIV-1 infection, by RT–qPCR. We selected the lncRNA CTB-119C2.1 with the most significant expression difference and the largest number of coexpressed genes for qRT–PCR verification. The results confirmed that the expression of the lncRNA CTB-119C2.1 was higher in PBMCs after ART treatment (Fig. 5A). Then, the coexpressed genes of the lncRNA CTB-119C2.1 were analyzed to reveal the potential function of this lncRNA (Fig. 5B). KEGG analysis of the associated genes showed that genes were most enriched in p53 signaling. Among which RAB3A and GADD45A have been shown to be associated with HIV infection (Fig. 5C).

**Table 2:** Subjects' age, sex, CD4+ T lymphocyte count, CD8+ T lymphocyte count, plasma viral load, ART regimen and duration.

Label	Gender	Age	Detection time	CD4 + T lymphocyte (count/µl)	CD8 + T lymphocyte (count/µl)	Plasma viral load (copies/ml)	ART regiment	ART duration (month)
b1	Male	19	2019/3/14	205	714	56,997	TDF + 3TC + EFV	
a1	Male	–	2019/10/11	297	600	< 20		7.5
b2	Male	25	2019/3/17	236	642	14,644	TDF + 3TC + EFV	
a2	Male		2019/10/14	458	610	< 20		7.5
b3	Male	27	2019/5/16	243	715	48,317	TDF + 3TC + EFV	
a3	Male		2019/11/12	509	1190	< 20		5.5
b4	Male	26	2019/6/14	444	692	5250	TDF + 3TC + EFV	
a4	Male		2019/9/12	482	522	< 20		3.0
b5	Male	24	2019/7/16	291	1040	75,746	TDF + 3TC + EFV	
a5	Male		2020/1/12	322	640	< 20		6.0
b6	Male	41	2019/7/20	266	1110	196,756	TDF + 3TC + EFV	
a6	Male		2020/1/15	514	920	< 20		6.0
b7	Male	22	2019/7/8	324	940	8530	TDF + 3TC + EFV	
a7	Male		2020/1/4	388	820	< 20		6.0
b8	Male	20	2019/7/12	324	530	3571	TDF + 3TC + EFV	
a8	Male		2020/1/10	602	710	< 20		6.0

## Discussion

In this study, we compared the expression profiles of lncRNAs before and after ART treatment and further analyzed their potential function. A total of 974 lncRNAs and 619 associated genes, whose expression levels became abnormal after HIV-1 infection and were restored to normal after ART treatment, were identified. We believe that these lncRNAs, along with their associated genes, may have a potential impact on HIV-1 replication.

LncRNAs have many functions in vivo, participating in the epigenetic, transcriptional, posttranscriptional, and translational regulation of genes and acting as vectors in biological processes^17^. Studies have found that the expression or functional abnormalities of lncRNAs are closely related to the occurrence of human diseases, including cancer^18,19^, neurodegenerative diseases^10^, and diabetes^20^. LncRNAs also play an important role in viral infection, and some studies have shown that lncRNAs are involved in the regulation of many biological processes during HIV infection^21^. RNA-seq is a quantitative and extremely sensitive technique for genome-wide transcriptome analysis based on sequencing. This technique and its derivative methods have been widely used to identify potential lncRNAs in several species^22^. In this study, we analyzed the expression changes of lncRNAs in PBMCs of healthy people and HIV/AIDS patients before and after ART treatment by RNA-seq.

Many genes showed significant abnormal expression patterns during HIV infection, and some of these genes were restored to normal expression after treatment. The upregulated genes were enriched in immune/inflammatory processes, while the downregulated genes were enriched in cell–cell/cell–matrix processes. Although the expression of inflammation-related genes decreased after treatment, there was still widespread inflammation in the infected patients. The expression levels of genes in cell–cell/cell–matrix processes decreased after treatment, which may be related to the persistence of the consequences of HIV-1 infection.

The PCA clustering results of identified lncRNAs showed significant expression differences during HIV infection and treatment. We found that a total of 974 abnormally expressed lncRNAs (268 upregulated lncRNAs and 706 downregulated lncRNAs) were restored to normal expression levels after treatment. Homeopathic regulation target gene analysis of the lncRNAs showed that RP11-685M7.3, AC002550.5, RP 11-290F5.1, RP 11-96D1.3, RP 5-1057120.4, and RP 11-972P1.10 have *cis*-regulation target genes. These lncRNAs may have a potential role in HIV infection and pathogenesis. For example, interferon regulatory factor 2 (IRF2), a *cis*-regulatory target gene of lncRNA RP 11–290F5.1, has been reported to be associated with HIV infection^23^. As the most representative members of the IRF family, IRF1 and IRF2 can be involved in a variety of biological processes, including inflammation, immune response, cell proliferation, and differentiation^24^. Previous studies have shown that a sequence homologous to ISRE, which is the binding site of IRF1 and IRF2, is in the 5ʹ LTR downstream of HIV-1. Deletion of the LTR-ISRE sequence leads to impaired LTR promoter activity and decreased synthesis of viral RNA and proteins^25^. In the absence of viral transactivator (Tat), IRF2 can bind to the LTR-ISRE sequence and drive LTR transcription^26^. The IRF2 gene is a cis-regulatory target of the lncRNA RP11-290F5.1, suggesting that this lncRNA may play a regulatory role in the process of HIV-1 infection.

Through coexpression analysis of differentially expressed lncRNAs and genes, transregulatory targets of lncRNAs were identified. The results indicated that the target genes participate in multiple biological processes, including the immune inflammatory response, angiogenesis, cell proliferation, and mitotic cell cycle, and these genes could be involved in the complications caused by HIV-1 infection^27–30^. For example, IL-1α, IL-10, CCL2, LMNA, and VCAM1, the target genes identified in this study, have been reported to be associated with HIV-1 infection and pathogenesis^31–33^. It is worthwhile to further explore the regulatory relationship between the lncRNAs and their target genes identified in this study. The lncRNA CTB-119C2.1, with the most significant differential expression pattern and the most coexpression-associated genes, was selected for qRT–PCR verification. Its qRT–PCR results were consistent with those of RNA-seq. The results indicated that the abnormally downregulated expression level of the lncRNA CTB-119C2.1 during HIV-1 infection increased after treatment. The following coexpression analysis showed that its associated genes were mainly involved in the p53 pathway and the signaling pathways responsible for the cell cycle and DNA replication, suggesting that the lncRNA CTB-119C2.1 may play an important role during HIV-1 infection and treatment. The detailed mechanism needs to be clarified in further studies^34–36^.

There are also some limitations in this study. First, the healthy control group was mostly female, this may introduce experimental bias in this study, further studies are needed to explore the influence of gender in HIV-1 infection. Second, only total PBMCs were investigated in this study, and further studies are needed to clarify the role of lncRNAs in specific cell types. Third, although the regulatory network of lncRNA-related genes has been obtained, more experiments are still needed to confirm the results. At last, the number of patients investigated in this study is small, and more samples should be included in the future to confirm the universality of the results.

## Conclusion

In conclusion, our study proved that HIV-1 infection can cause abnormal expression of lncRNAs and their associated genes, some of which can be restored to normal after effective ART treatment. Bioinformatic analysis revealed that lncRNAs RP11–290F5.1 and CTB-119C2.1 may play a key role in HIV-1 infection and pathogenesis, which is worthy of further exploration of the specific mechanism.

## Methods

### Patients

We recruited six HIV/AIDS patients who were treated and followed up at the AIDS Center of Zhongnan Hospital of Wuhan University from December 2018 to December 2019. HIV/AIDS Diagnostic Criteria Reference to the Guidelines for the Diagnosis and Treatment of AIDS, 3rd Edition (2015 Edition). Additionally, the data of six healthy volunteers who underwent physical examinations in the physical examination center of Zhong Nan Hospital of Wuhan University during the same period were collected. The initial screening for HIV-1 antibody was negative, and all routine indicators were normal.

All the study subjects were 18 years or older. The subjects were divided into two groups: the health group (6 healthy volunteers) and the treatment group (6 persons included in pre- and post-treatment data). All the cases in this study were from outpatients and inpatients of the Department of Infectious Diseases, Zhongnan Hospital of Wuhan University. The exclusion criteria are as follows: patients with acute infection of HIV-1; patients with comorbid conditions such as obesity, cardiovascular disease, diabetes mellitus, cancer and mental system disease; patients with other virus infection such as HBV, HCV and CMV; patients with opportunistic infections such as PCP, fungal infection, and tuberculosis; patients with intravenous drug uses and alcohol abuse. Peripheral blood monocytes were separated by density gradient centrifugation. They were cryopreserved in fetal calf serum (Gibco) containing 10% DMSO (Sigma) and stored in liquid nitrogen. The study scheme was approved by the Ethics Committee of Zhong Nan Hospital of Wuhan University, and all participants signed the relevant informed consent form. All methods were performed in accordance with the relevant guidelines and regulations.

### Laboratory testing

A total of 18 whole blood samples from 12 participants were collected. For flow cytometric analysis, whole blood was collected in k3 EDTA vials. Whole blood was permeabilized and fixed using Cytofix/Cytoperm (BD Pharmingen, San Jose, CA, USA) according to the manufacturer’s protocol, followed by staining for 20 min at room temperature in the dark with a cocktail of antibodies, including anti-CD4-APC, anti-CD8-PE, and anti-CD3-PerCP (BD Pharmingen, CA, USA). After staining, RBCs were lysed using BD FACS lysing solution (BD Pharmingen, San Jose, CA, USA) according to the manufacturer’s instructions. More than 50,000 cells were acquired for flow cytometric analyses on a BD FACSCaliber, and the results were analyzed using TreeStar FlowJo software version 8.8.7. HIV viral load was determined using NucliSens Easy Q HIV-1 v2.0 (bioMerieux, Iyon, France), with a limit of detection of 20 copies/ml.

### RNA isolation and quality control

Total RNA was extracted using the Qiagen RNeasy kit (QIAGEN), and during the purification process of the sample, DNA enzyme (QIAGEN) was used for on-column digestion of the DNA. RNA quality and concentration were measured using an Agilent 2100 biological analyzer.

### RNA sequencing

Ribosomal RNA molecules (rRNA) were eliminated using Ribo-Zero rRNA removal kits (Illumina). RNA sequencing libraries were constructed using the TruSeq Stranded Total RNA Library Prep Kit (Illumina) according to the manufacturer's instructions, and purified library products were evaluated using 2200 Tapestation (Agilent Technologies) and Qubit®2.0 (Thermo Fisher Scientific). Finally, single-stranded DNA molecules were clustered and sequenced for 150 cycles on an Illumina HiSeq 3000 system (Illumina)(“Supplementary information”).

### Data clean process

The raw reads were trimmed of low-quality bases using a FASTX-Toolkit (v.0.0.13; http://hannonlab.cshl.edu/fastx_toolkit/). Then, the clean reads were evaluated using FastQC (http://www.bioinformatics.babraham.ac.uk/projects/fastqc).

### Read alignment and differentially expressed gene (DEG) analysis

Clean reads were aligned to the human GRch38 genome by tophat2^37^, allowing 4 mismatches. Uniquely mapped reads were used to calculate the read number and reads per kilobase of exon per million fragments mapped (RPKM) for each gene. The expression levels of genes were evaluated using RPKM. The software edgeR^38^, which is specifically used to analyze the differential expression of genes, was applied to screen the RNA-seq data for DEGs. The results were analyzed based on the fold change (FC ≥ 2 or ≤ 0.5) and p value (p value ≤ 0.05) to determine whether a gene was differentially expressed.

### LncRNA prediction and direction identification

To systematically analyze the lncRNA expression pattern, we used a pipeline for lncRNA identification similar to that previously reported^39^, which was constructed based on cufflinks software^40^. All steps of the pipeline have been shown in Fig. 1A.

### Coexpression analysis

To explore the regulatory mode between lncRNAs and mRNAs, we calculated the Pearson’s correlation coefficients (PCCs) between them with a correlation of 0.6 and a p value of 0.05. Then, the distance between lncRNAs and genes (10 kb) was used as the threshold to determine the cis- and trans-regulatory relationships. A distance between lncRNAs and genes less than 10 kb was defined as cis regulation, and a distance greater than 10 kb or on other chromosomes was considered trans regulation.

### Predicting targets of *trans*- and *cis*-acting lncRNA

We first identified all the coexpression pairs of lncRNAs and genes in each sample by calculating Pearson’s correlation coefficients (|r| > 0.6 and p value < 0.05) between the expression levels of differential lncRNAs and all genes. Then, with a threshold distance of 10 kb between lncRNA and gene pairs, cis-acting genes were identified. The other lncRNA and gene pairs with distances greater than 10 kb or in the different chromosomes were regarded as trans-acting pairs.

### Functional enrichment analysis

To sort out functional categories of genes, Gene Ontology (GO) terms and Kyoto Encyclopedia of Genes and Genomes (KEGG) pathways were identified using the KOBAS 2.0 server^41–44^. Hypergeometric tests and Benjamini–Hochberg FDR controlling procedures were used to define the enrichment of each term. Reactome (http://reactome.org) pathway profiling was also used for functional enrichment analysis of the sets of selected genes. The adjust p values ≤ 0.05 was considered to be significant.

### Real time quantitative PCR validation

We recruited 8 HIV-1 infected patients for the validation study, all these patients were not participant in the initial screening (Table 2), the exclusion criteria of these 8 HIV-1 infected patients were the same as previous description. A total of 16 whole blood samples (5 ml per piece) were collected from 8 participants, and PBMCs were isolated. Total RNA was extracted using the RNeasy Mini Kit (Qiagen). The RNAs were reverse transcribed with a reverse transcription kit (riboSCRIPTTM mRNA/lncRNA qRT–PCR Starter Kit (20 T RT + 60 T PCR) (Guangzhou RuiboBio Co., Ltd.). Applied Biosystems™ SYBR™ Green master mixes (Thermo Fisher Scientific) were used for RT–qPCR. GADPH serves as an internal reference gene. The relative expression of lncRNAs was calculated by the 2^−△△Ct^ method. Statistical analysis of qRT–PCR was performed by GraphPad Prism 7 (GraphPad Software, Inc., La Jolla, CA, USA), and comparison between the two samples was performed by t-test.

### Other statistical analysis

Principal component analysis (PCA) analysis was performed by the R package factoextra (https://cloud.r-project.org/package=factoextra) to show the clustering of samples with the first two components. After normalizing the reads by FPKM (fragments per kilobase of exon model per million mapped fragments) of each gene in samples. The pheatmap package (https://cran.r-project.org/web/packages/pheatmap/index.html) in R was used to perform clustering based on Euclidean distance. Student’s t-test was used for comparisons between two groups. Cytoscape (v3.5.1) was used to display the network of lncRNAs and mRNAs. PPI analysis was performed by an online website (https://string-db.org/).

### Ethics approval and consent to participate

Ethical approval was granted by the Medical Ethics Committee, Zhongnan Hospital of Wuhan University. The reference number is 2019-119.

### Supplementary Information

Supplementary Information 1.

Supplementary Information 2.

## Data Availability

All relevant data and materials from this study are included in this published article. The datasets generated and/or analyzed during the current study are available in the [GSE235038] repository, [https://www.ncbi.nlm.nih.gov/geo/query/acc.cgi?acc=GSE235038].

## Abbreviations

LncRNAs: Long noncoding RNAs
HIV: Human immunodeficiency virus
ART: Antiretroviral therapy
PBMCs: Peripheral blood mononuclear cells
IRF2: Interferon regulatory factors 2
RNA-seq: RNA-sequencing
AIDS: Acquired immunodeficiency syndrome
cART: Combination antiretroviral therapy
RPKM: Reads per kilobase of exon per million fragments mapped
PCCs: Pearson’s correlation coefficients
GO: Gene Ontology
KEGG: Kyoto Encyclopedia of Genes and Genomes
PCA: Principal component analysis
